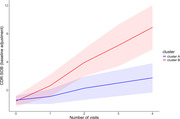# Subtypes of Longitudinal Progression Trajectories Among Cognitively Impaired Older Adults with A+N+ Biomarkers: Trajectory Clustering Analysis

**DOI:** 10.1002/alz.095431

**Published:** 2025-01-09

**Authors:** Sangyong Park, Min Soo Byun, Dahyun Yi, Hyejin Ahn, Evgeny J. Chumin, Gijung Jung, Kyung Tae Kim, Hyeji Choi, Yoon Hee Kim, Yu Kyeong Kim, Yun‐Sang Lee, Koung Mi Kang, Chul‐Ho Sohn, Jun‐Young Lee, Shannon L. Risacher, Olaf Sporns, Andrew J. Saykin, Kwangsik Nho, Dong Young Lee

**Affiliations:** ^1^ Seoul National University Hospital, Seoul Korea, Republic of (South); ^2^ Seoul National University College of Medicine, Seoul Korea, Republic of (South); ^3^ Seoul National University Medical Research Center, Seoul Korea, Republic of (South); ^4^ Seoul National University, Seoul Korea, Republic of (South); ^5^ Indiana University School of Medicine, Indianapolis, IN USA; ^6^ SMG‐SNU Boramae Medical Center, Seoul Korea, Republic of (South); ^7^ Indiana University, Bloomington, IN USA

## Abstract

**Background:**

We investigated heterogeneities in clinical progression trajectories among cognitively impaired (CI) older adults who were positive for both beta‐amyloid (Aβ) and neurodegeneration biomarkers of Alzheimer’s disease (AD) using trajectory clustering analysis. We then compared clinical and neuroimaging variables across clusters with different clinical trajectories.

**Method:**

CI older adults, consisting of individuals with mild cognitive impairment (MCI) or mild AD dementia were recruited from the Korean Brain Aging Study for the Early Diagnosis and Prediction of Alzheimer’s disease (KBASE). All participants underwent comprehensive clinical assessment, and multi‐modal neuroimaging including ^11^C‐PiB PET, ^18^F‐FDG PET, and MRI with resting‐state functional MRI (fMRI). Among them, participants who were both amyloid positive (A+) and neurodegeneration positive (N+), including those with hypometabolism and cortical thinning in AD‐vulnerable regions, as well as hippocampal atrophy, were included. A subset of participants underwent ^18^F‐AV1451 PET to measure brain tau deposition. Group‐based trajectory modeling (GBTM) using the Clinical Dementia Rating (CDR)‐Sum of boxes (SOB) measured at baseline and longitudinal follow‐up up to four years, was used to identify clusters among A+N+ CI participants.

**Result:**

A total of 86 A+N+ CI individuals were included for the final analysis. A GBTM, based on longitudinal CDR‐SOB, identified two clusters with different trajectories: Cluster A (N = 54 [62.8%]) with slow progression and Cluster B (N = 32 [37.2%]) with rapid progression (Figure 1). No significant differences among age, sex, educational years, clinical diagnosis, global CDR, and APOE e4 carrier status were observed between the two clusters at baseline. These two clusters did not differ regarding global tau deposition and Braak Stages in a subset of participants (N = 34). However, at baseline, network segregation measure for the whole cortex and sensory‐motor network, and functional connectivity (FC) within the sensory‐motor network, differed between the two clusters after adjusting for age, sex, and education.

**Conclusion:**

Our study identified two clusters with heterogeneous clinical progression trajectories even among CI older adults who exhibited both Aβ and neurodegeneration biomarkers. Further studies are necessary to elucidate the relationship between resting‐state FC measures and AD subtypes with different clinical trajectories.